# Extent of Height Variability Explained by Known Height-Associated Genetic Variants in an Isolated Population of the Adriatic Coast of Croatia

**DOI:** 10.1371/journal.pone.0029475

**Published:** 2011-12-27

**Authors:** Ge Zhang, Rebekah Karns, Guangyun Sun, Subba Rao Indugula, Hong Cheng, Dubravka Havas-Augustin, Natalija Novokmet, Dusko Rudan, Zijad Durakovic, Sasa Missoni, Ranajit Chakraborty, Pavao Rudan, Ranjan Deka

**Affiliations:** 1 Human Genetics Division, Cincinnati Children's Hospital, Cincinnati, Ohio, United States of America; 2 Center for Genome Information, Department of Environmental Health, University of Cincinnati, Cincinnati, Ohio, United States of America; 3 Institute for Anthropological Research, Zagreb, Croatia; 4 Center for Computational Genomics, Institute of Investigative Genetics, University of North Texas Health Science Center, Forth Worth, Texas, United States of America; Central China Normal University, China

## Abstract

**Background:**

Human height is a classical example of a polygenic quantitative trait. Recent large-scale genome-wide association studies (GWAS) have identified more than 200 height-associated loci, though these variants explain only 2∼10% of overall variability of normal height. The objective of this study was to investigate the variance explained by these loci in a relatively isolated population of European descent with limited admixture and homogeneous genetic background from the Adriatic coast of Croatia.

**Methodology/Principal Findings:**

In a sample of 1304 individuals from the island population of Hvar, Croatia, we performed genome-wide SNP typing and assessed the variance explained by genetic scores constructed from different panels of height-associated SNPs extracted from five published studies. The combined information of the 180 SNPs reported by Lango Allen el al. explained 7.94% of phenotypic variation in our sample. Genetic scores based on 20∼50 SNPs reported by the remaining individual GWA studies explained 3∼5% of height variance. These percentages of variance explained were within ranges comparable to the original studies and heterogeneity tests did not detect significant differences in effect size estimates between our study and the original reports, if the estimates were obtained from populations of European descent.

**Conclusions/Significance:**

We have evaluated the portability of height-associated loci and the overall fitting of estimated effect sizes reported in large cohorts to an isolated population. We found proportions of explained height variability were comparable to multiple reference GWAS in cohorts of European descent. These results indicate similar genetic architecture and comparable effect sizes of height loci among populations of European descent.

## Introduction

Heritability of normal variation in human height is estimated to be ∼80%–90% [1]–[4], much of which is attributed to inherited factors [5]. Genome-wide association studies (GWAS) and gene-centric analyses have uncovered over 200 single nucleotide polymorphisms (SNPs) associated with human height, but these variants explain only 2∼10% of the phenotypic variance [6]–[12]. Arguing that a large fraction of missing heritability could potentially stem from the large number of height-related variants with small effect sizes, a recent study analyzed all genome-wide SNPs simultaneously and reported that 45% of genetic variation in height could be explained by common variants [13]–[15] without specifying their individual contributions.

The above mentioned GWAS and meta-analysis were based on large cohorts primarily of European descent sampled from Europe and North America, although a few have included other ethnicities [6], [12]. In this study, we have assessed the variance explained by known height-associated loci in a relatively isolated island population from the Adriatic coast of Croatia with limited admixture and homogeneous genetic background. Rather than conducting a discovery study, our purpose was to evaluate whether the effects of genetic variants uncovered in larger, heterogeneous study populations are portable to smaller, homogeneous populations. Specifically, we conducted a GWAS using a panel of 500K SNPs in a sample of >1300 adults to identify height-associated variants and estimated variance explained using genetic scores that combine information from panels of known loci from published GWAS [6]–[9], [11]. Our study showed that comparable variance may be explained by previously reported height-associated GWAS variants, if the comparison population is of European decent, and that the associated loci and effect sizes of previously identified loci are comparable in our population.

## Methods

### Study Population

The study population was derived from a genetic study of metabolic syndrome [16], [17]. Briefly, 1445 men and women were recruited from Hvar, a middle Dalmatian island on the eastern Adriatic coast of Croatia. The present day Adriatic islanders are primarily of Slavic descent, who had emigrated from the mainland before the 18th century and remained relatively isolated since that time with attributes of relative homogeneous genetic, cultural and environmental background [18], [19]. Blood samples and anthropometric data were collected in two field seasons of May 2007 and May 2008. For this study, we used the data on 1304 participants for whom genome-wide SNP data and height measurements were available. The study was approved by the Ethics Committee of the Institute for Anthropological Research in Zagreb, Croatia and the Institutional Review Board of the University of Cincinnati. Written informed consent was obtained from all participants.

### Genotyping and imputation

Genome-wide SNP genotyping was performed using the Affymetrix Human SNP Array 5.0 following the manufacture's protocol. Genotype calls were determined using the CRLMM algorithm [20], [21] among chips that passed the DM QC call rate (>0.86). Following further QC filtering of the genotype data (MAF>0.02, HWE P>0.0001, call rate>95%) using the check.marker function implemented in GenABEL [22], we obtained a cleaned data set of 344,512 SNPs in 1304 samples (565 males and 739 females). On this data set, we performed genotype imputation using MACH [23] and the reference haplotype data from the Phase II CEU HapMap [24], yielding genotype data on 2.5 million SNPs.

### Genome-wide association analyses

The study sample included 533 unrelated and 771 related individuals from 238 families. Therefore, two different methods were used to account for the relatedness – the genomic control (GC) [25], [26] and the measured genotype (MG) approach [27]. In GC, all samples were treated as unrelated and the inflation factor (λ) was estimated using the median method [28]. In the MG approach, the kinship matrix was estimated from all autosomal SNPs instead of pedigree data. To assess the empirical performance of the two methods, we examined how many of the previously identified height loci (http://www.genome.gov/gwastudies/) could be recovered from the top 100 significant regions (defined as 250 kb on either side of associated SNPs) using each of these two methods. Standardized height adjusted for age and gender and their interaction term was used in all analyses. P-values and effect size estimates assuming additive allelic effect are reported throughout the manuscript. All statistical analyses were performed in R v2.12 and the GenABEL package (v.1.6) [22].

### Variance explained by identified loci

To assess the overall fitting of previously identified height-associated loci in our study sample, we estimated the percentage of variance explained using genetic scores that combine information from different panels of known loci reported in previous studies. The unweighted genetic score was computed as an individual's number of height-increasing alleles, and the weighted genetic score was constructed as 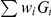; 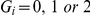 indicates the number of reference alleles for specific SNP, 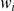 is the appropriate weight proportional to previously reported estimates of allelic effect (measured as beta-coefficient or log transformed odds ratio). The variance explained (r^2^) was then estimated based on a linear regression model using the scores as predictor and age-, gender-, and their interaction term adjusted height residuals as outcome.

We estimated and compared the variance explained based on different panels of height-associated SNPs and their effect size estimates extracted from published large-scale GWAS, which included 1) 180 genome-wide significant SNPs reported by Lango Allen et al. [11], 2) height-associated SNPs reported by the three GWA studies published in the 2008 May issue of Nature Genetics [6]–[8], and 3) 54 height SNPs used by Aulchenko et al [9], which were derived from the three aforementioned GWAS [6]–[8].

## Results

Height was normally distributed in both males (N = 565) and females (N = 739), and males (177.7±7.5 cm) were significantly taller than females (164.4±7.1 cm). Regression analysis showed that adult height was negatively associated with age in both genders with a larger age-effect in females. Estimated height decrease per year in females was 0.239 cm (P = 9.05×10^−55^, *r* ^2^ = 28.0%) and in males was 0.195 cm (P = 3.50×10^−24^, *r* ^2^ = 16.6%, Figure S1).

**Figure 1:**
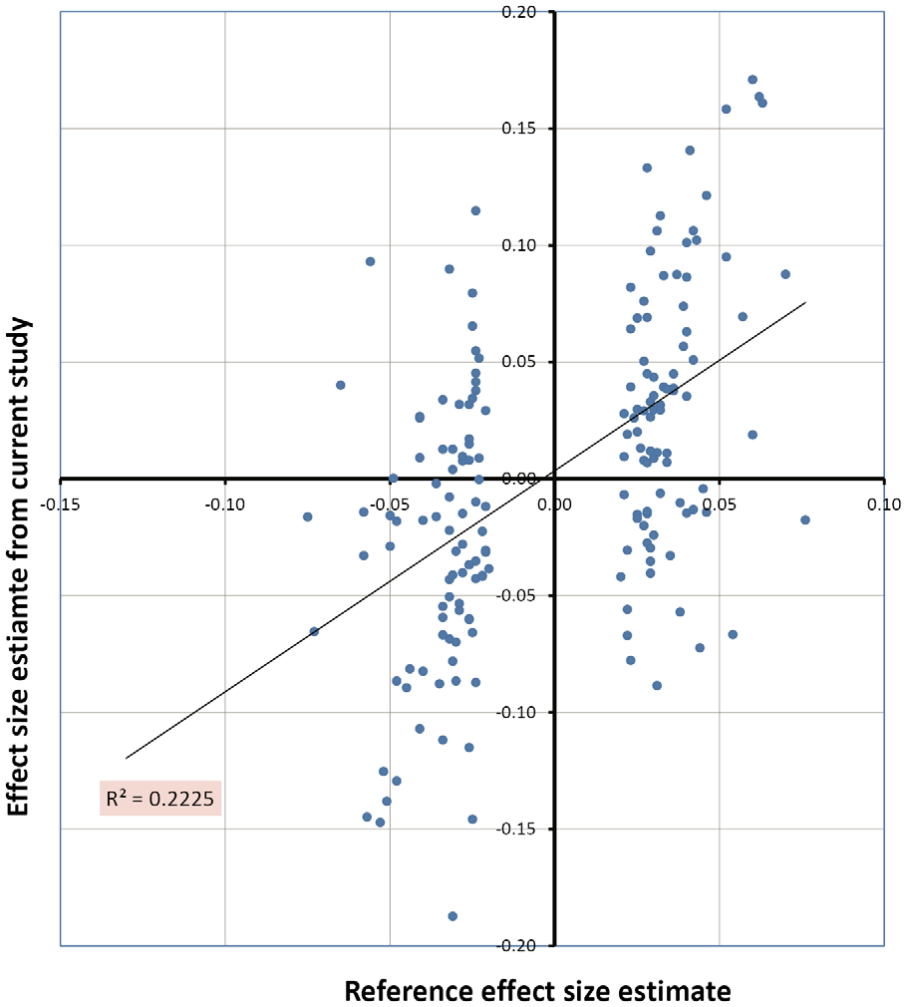
Effect size estimates of the 180 height SNPs were correlated between our study and the reference study by Lango Allele et al.

**Figure 2:**
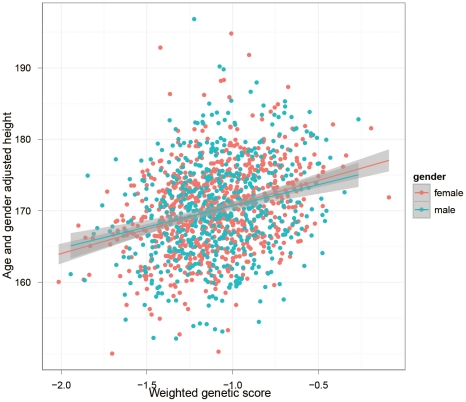
Correlation between height and weighted genetic scores in males and females.

Genetic scores based on the five published studies are shown in Table 1. The allele frequencies of the 180 genome-wide significant SNPs reported by Lango Allen et al. [11] in our samples were similar to the original report (Figure S2). The correlation of effect size estimates of these SNPs between our study and the original report (Stage 1+2) was highly significant although not perfect (Pearson ρ = 0.472, P = 2.32×10^−11^). The effect size estimates of 58 SNPs were in different directions (points in the 2nd and 4th quadrants in Figure 1). After removing these 58 SNPs, Pearson's ρ increased to 0.851 for the remaining 122 SNPs. However, heterogeneity test did not show significant differences in estimates of effect sizes between our study and that of Lango Allen et al. [11]. Though 12 of the 180 SNPs showed nominally significant heterogeneity (P<0.05) none survived adjustment for multiple testing (a full list of these 180 SNPs are provided in the Table S1). As expected, based on the central limit theorem, the genetic scores were normally distributed (Figure S3). The weighted genetic score and adjusted height were significantly (P<1×10^−16^) correlated (Figure 2). Combining the information from the 180 reference SNPs the explained variance was 7.94%, which was at the lower bound of the range of variance explained reported by Lango Allen et al. – 10.5% (range 7.9–11.2%) [11]. As reflected in Figure 2, the correlation of genetic score with adjusted height was higher in females than males with a larger fraction of variance explained (10.09% vs 5.87% in females and males respectively). The unweighted genetic scores explained a lower fraction (5.45%) of the overall variance.

**Table 1:** Genetic scores based on different reference studies.

Reference studies(Authors)	Data Table#	# SNPs$	Reference effect sizes&		ρ_eff_ *	Variance explained (%)	
			Source	Sample size		Unweighted	Weighted
Nature 467:832-8 [11]	Suppl Table 1	180	Stage 1	133653	0.455	5.45	7.79
Lango Allelen et al.			Stage 2	50074	0.497	5.45	7.95
			Stage 1+2	183727	0.472	5.45	7.94
			Females	110489	0.461	5.45	7.87
			Males	73238	0.486	5.45	7.80
Nat Genet 40:609-15 [6]	Table 1 and 2	57	Iceland	25174	0.523	3.35	3.58
Gudbjartsson et al.			Holland	2876	0.550	3.35	3.72
			US Cau	1770	0.448	2.62	2.69
			US Afr	1148	0.199	0.81	1.01
Nat Genet 40:584-91 [7]	Table 1	29	Meta	15821	0.492	3.50	3.48
Lettre et al.			Follow-up	19990	0.577	1.43	3.38
			Combined	35811	0.539	3.50	3.85
			USHT*	2189	0.639	3.19	4.62
Nat Genet 40: 575-83 [8]	Table 1	20	Female	na	0.657	3.18	3.20
Weedon et al.			Male	na	0.732	3.18	3.77
Eur J Hum Genet 17:1070-5	Suppl Table 1	54	Add, single	5748	0.608	4.75	4.83
Aulchenko et al.			Genotype	5748	-	4.07	4.28

# The table number in the reference study, from where the list of height-associated SNPs and their effect size estimates were extracted to build the genetic scores.

$ The number of height-associated SNPs reported by the reference study.

& Individual reference studies report multiple effect sizes estimated from multiple sources with different sample sizes. Based on these estimates, different genetic scores can be constructed.

*The Pearson correlation between the effect sizes extracted from the original report and estimated from our samples.

The weighted genetic scores constructed using the SNPs and effect size estimates reported by the three Nature Genetics studies [6]–[8] explained 3∼4% of the height variance in our study sample. The study by Gudbjartsson et al. [6] included an African American cohort (n = 1,148) and the variance explained by the genetic scores built on the effect sizes estimated from this group was significantly smaller (P<0.001) than the scores based on the effect sizes estimated from other groups of European descent (Icelandic, Dutch or the Europeans Americans), which may reflect effect size differences among diverse populations.

In addition to testing height as a quantitative variable, Lettre et al. [7] evaluated their identified SNPs in a height ‘case-control’ sample (the USHT tall-short panel with subjects selected from tails of the height distribution) and reported allelic effects using odds ratios (ORs). Our effect size estimates correlated well with the log transformed ORs (Pearson ρ = 0.639) and the genetic scores weighted by these log transformed ORs explained even higher variance (4.62%) than the genetic scores weighted by the beta value estimates (Table 1).

In addition, we estimated the variance explained by the panel of 54 SNPs analyzed by Aulchenko et al. [9], which were identified by the three aforementioned GWAS [6]–[8]. The weighted and unweighted genetic scores based on these 54 SNPs explained slightly larger variances than the scores based on any of the individual studies (Table 1). This study also reported genotypic effect sizes, which enabled us to construct weighted genotype scores without the assumption of allelic additivity, though the variance explained by this genotypic score was similar to that obtained by weighted genetic scores assuming additive allelic effects.

The reported height-associated SNPs that overlapped across studies may capture more genuine effects than those obtained from a single study. Therefore, we tested whether the variance explained can be increased by including multiple genetic scores as multi-dimensional predictors. By including the scores with largest variance explained from each study, the explained variance was increased to 10.21%.

We also performed genome-wide association analysis in order to replicate previous findings. As indicated by QQ-plots (Figure S4), both GC and MG appropriately controlled type I error rates. The genome-wide single-locus test statistics were inflated compared to the null distribution with an estimated inflation factor λ = 1.41. As expected this estimate was the highest among all metabolic traits we studied (unpublished data) since the inflation factor is larger for traits with higher heritability and substantial inflation is likely attributable to the large number of SNPs associated with height [29].

**Figure 3:**
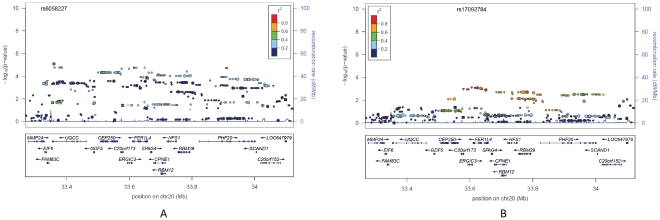
LocusZoom plot of the GDF5-UQCC region before (A) and after (B) conditioning. The most significant SNP was rs17092784 after conditioning on rs6058227.

The P-values obtained by GC and MG were highly correlated (Pearson's ρ = 0.766). Interestingly, the GC method recovered 9 previously known loci (Table 2 and Figure S5) compared to 4 recovered by the MG approach, three of which were in the 9 GC regions. Due to our limited sample size, however, none of these loci reached genome-wide significance (P<5×10^−8^). The 9 GC regions include several well known height loci, including EFEMP1, UQCC and HMGA2. Of particular interest is the UQCC locus (also known as the GDF5-UQCC region; Figure 3A). This region encompasses approximately 850 kb, covering more than 20 genes, and has a relatively low recombination rate. The most significant signal was observed on SNP rs6058227 (P = 8.21×10^−6^); the minor allele (T) was associated with an average increase of height 1.93 cm. Re-analysis of the region conditional on rs6058227 failed to completely eliminate the signals of the other SNPs, suggesting the possibility of multiple causal variants within the region (Figure 3B). Similar evidence for allelic heterogeneity was also reported by Lango Allen et al., and the presence of multiple functional variants could be a potential source of missing heritability [11].

**Table 2:** Nine replicated height-associated loci identified by GC approach.

No	Regions#				Index SNPs$			GWAS SNPs&	Genes
	chr	start	end	length	rs_id	MAF	P-value		
**1** *	2	55822780	56080130	257351	rs6730839	0.25	1.53×10^−6^	rs3791679	EFEMP1
								rs3791675	
**2** *	20	33267822	34124695	856874	rs6058227	0.13	8.21×10^−6^	rs6060369	UQCC
								rs6088813	
								rs6060373	
								rs2236164	
								rs6088792	
3	5	152756659	153018678	262020	rs7737904	0.03	2.12×10^−5^	rs12658202	GRIA1
**4** *	12	64588210	64702663	114454	rs1042725	0.49	5.21×10^−5^	rs8756	HMGA2
								rs1042725	
5	6	130271009	130327351	56343	rs10457532	0.24	1.22×10^−4^	rs6899976	L3MBTL3
								rs6569648	
6	12	92723646	92899954	176309	rs10444517	0.06	1.24×10^−4^	rs11107116	CRADD
								rs3825199	
7	3	135631761	135778576	146816	rs9866359	0.14	1.44×10^−4^	rs10935120	ANAPC13
8	7	28128599	28246163	117565	rs849315	0.12	1.62×10^−4^	rs1635852	JAZF1
								rs849141	
9	12	101541161	101630322	89162	rs833718	0.50	1.94×10^−4^	rs5742692	IGF1
								rs1520223	

# Each significant region was selected starting with a “seed” SNP (P<5×10^−4^) and was extended to adjacent SNPs with (P<5×10^−3^) within 25 kb. The chromosomal positions were based on NCBI Genome Build 36.3.

$ Index SNPs were the most significant SNP observed in each region.

& GWAS SNPs were known height-associated SNPs within or near (<250 kb) the region (extracted from GWA catalog: http://www.genome.gov/gwastudies/).

*These three regions were also in the top 100 regions identified by MG approach.

## Discussion

We presented an estimation of variance explained by previously reported height-associated loci in a relatively isolated population based on a genome-wide scan of 2.5 M genotyped and imputed SNPs in 1304 individuals. Given our relatively small sample size, we did not aim to identify novel loci; instead, our purpose was to evaluate whether height-associated SNPs identified by GWA studies in large population cohorts share similar effects in an isolated and homogeneous population.

We evaluated the percentage of variance explained by genetic scores based on previously reported panels of height-associated SNPs and their effect size estimates. Under the assumption of additive effect between loci, the weighted genetic score captures the majority of genetic effects at multiple loci. The corresponding explained variance can be used as an indicator of the overall agreement of the effect size estimates between reference studies (from which the effect sizes/weights were developed) and our current study. The results indicate that a comparable fraction of variance can be explained in our samples if the genetic score was constructed from a population of European descent. In our sample the variance explained by the 180 height-associated SNPs reported by Lango Allen et al. [11] was 7.94%, which was within the range (7.9–11.2%) of the original report, though at the lower end. The higher percentage of variance explained by the weighted scores compared to the unweighted scores (5.45%) also suggested that the effect sizes estimated from meta-analysis of multiple cohorts of European descent can be applied to our relatively isolated population, which is also of European descent. At the individual SNP level, we did not detect significant differences in effect size estimates between our study and the reference study. Altogether, these observations indicate similar genetic architecture (i.e. associated loci and their effect sizes) of height in the same continental populations.

Using the SNPs reported by the three Nature Genetics studies [6]–[8], the percentages of variance explained were around 3∼4%, which were within the range of the original reports. We also found that the log transformed odds ratios estimated from extreme “case/control” samples [7] provided accurate estimates of real effect sizes, illustrated by the larger variance explained compared to scores using beta coefficients estimated from unascertained population-based cohorts with larger sample sizes. The significantly lower variance explained using the genetic scores based on effect size estimates from US African samples demonstrated substantially different effect size estimates between distantly related populations. Since our sampled individuals contained familial relatedness, we tested two methods to account for relatedness. The measured genotype (MG) approach uses a mixed model to quantify the polygenic effect and the fixed effect (association) simultaneously and is a robust and powerful method for the analysis of quantitative traits in related samples [30]. Genomic control (GC) is a simpler and computationally efficient method originally developed to control the inflated false positive rate introduced by population stratification [26], [28] and has been recently applied to account for relatedness in association tests [31]. The GC method corrects the false positive rate by adjusting test statistics using a uniform inflation factor and it ranks significant findings in the same order as conventional association tests that do not account for relatedness.

Our results indicate that the empirical performance of the GC method was better than the MG approach by recovering a larger number of previously-identified height loci among the top ranking regions. This implied that the P-values reported by GC or association tests ignoring relatedness may better reflect the real order of significance than the complex approach of MG. Recent simulation studies indicate that GC or conventional tests are appropriate methods to study genetic association in randomly ascertained, moderate-size pedigrees for both quantitative [32] and qualitative traits [33]. However, for polygenic traits with high heritability (such as height), GC may be over-conservative since all SNPs, a substantial fraction of which are truly associated with the trait, are used to estimate the inflation factor [29], [32]. The inflation factor (λ = 1.41) for height observed in current analysis was the highest among all traits we studied in the same population sample (data not shown). This high estimate is likely explained by high heritability and the large number of associated SNPs rather than by population stratification or relatedness; therefore rendering the reported association P-values (Table 2) conservative.

Of the 9 height-associated loci described in Table 2, several were among the top of the significant lists in multiple GWAS; for example, HMGA2 [34] and GDF5-UQCC [35] were the first two identified height loci and were widely replicated in multiple studies [6]–[8]. The EFEMP1 locus was first reported by Weedon et al. [8] and Gudbjartsson et al. [6] and was subsequently replicated in Australians of European descent [36] and Asian populations [37]–[39]. Four other gene regions, L3MBTL3 [6], [11], CRADD [6], [8], JAZF1 [10], [40], and ANAPC13 [8] were also reported in European GWA studies. Of note is the GRIA1 locus, which was previously reported in a GWAS in another Adriatic island population [41]. Though the IGF1 (insulin-like growth factor 1) pathway is integral to growth and mutations in IGF1 usually result in growth retardation [42], candidate gene studies testing for associations between common variants of IGF1 and height variation have been inconclusive [43]–[45]. Two recent GWAS [38], [39] have, however, confirmed IGF1 as a height-associated locus in Asian populations. However, this locus has not been identified in European GWA studies with even much larger sample sizes, suggesting weak statistical powers due to differences in allele frequencies or linkage disequilibrium patterns between populations.

As in the study by Lango Allen et al. [11], through conditional analysis we found evidence supporting allelic heterogeneity in many height-associated regions. In the GDF5-UQCC region, for example, our most significant SNP (rs6058227) was not in close linkage disequilibrium with six index SNPs reported in previous GWA studies and analysis conditional on rs6058227 could not eliminate the significances of the adjacent SNPs in that region. These all are highly suggestive of multiple effective SNPs in this region. This observation again highlighted that allelic heterogeneity might be a common feature of polygenic loci and identification of additional functional variants beyond the lead SNPs may increase the proportion of variance explained for complex traits [11], [46].

In summary, we have evaluated the portability of height-related loci and their estimated effect sizes reported in large cohorts to an isolated population with a homogeneous genetic background. We found proportions of explained height variability were comparable to multiple reference GWAS in populations of European descent. These results indicate similar genetic architecture and comparable effect sizes of height loci among populations of European descent.

## Supporting Information

Figure S1 **Correlation of height with age in males and females.**
(TIF)

Figure S2 **Minor allele frequencies of the 180 height-associated SNPs were highly correlated between our study and the reference study by Lango Allen et al** [**11**] **.**
(TIF)

Figure S3 **Genetic scores follow normal distribution.**
(TIF)

Figure S4 **QQ plot of GC (A) and MG (B) association test.**
(TIF)

Figure S5 **Manhattan plot (the red bars indicated the nine replicated GWA regions).**
(TIF)

Table S1 **Frequencies and effect sizes of the 180 height-associated SNPs reported by Lango Allelen et al. [** ***Nature*** **467 (7317): 832-8].**
(PDF)
