# Supplementary figures and images for: Extent of Height Variability Explained by Known Height-Associated Genetic Variants in an Isolated Population of the Adriatic Coast of Croatia
Source: PLoS One. 2011 Dec 27;6(12):e29475. doi: 10.1371/journal.pone.0029475 (PMC3246488; doi:10.1371/journal.pone.0029475)

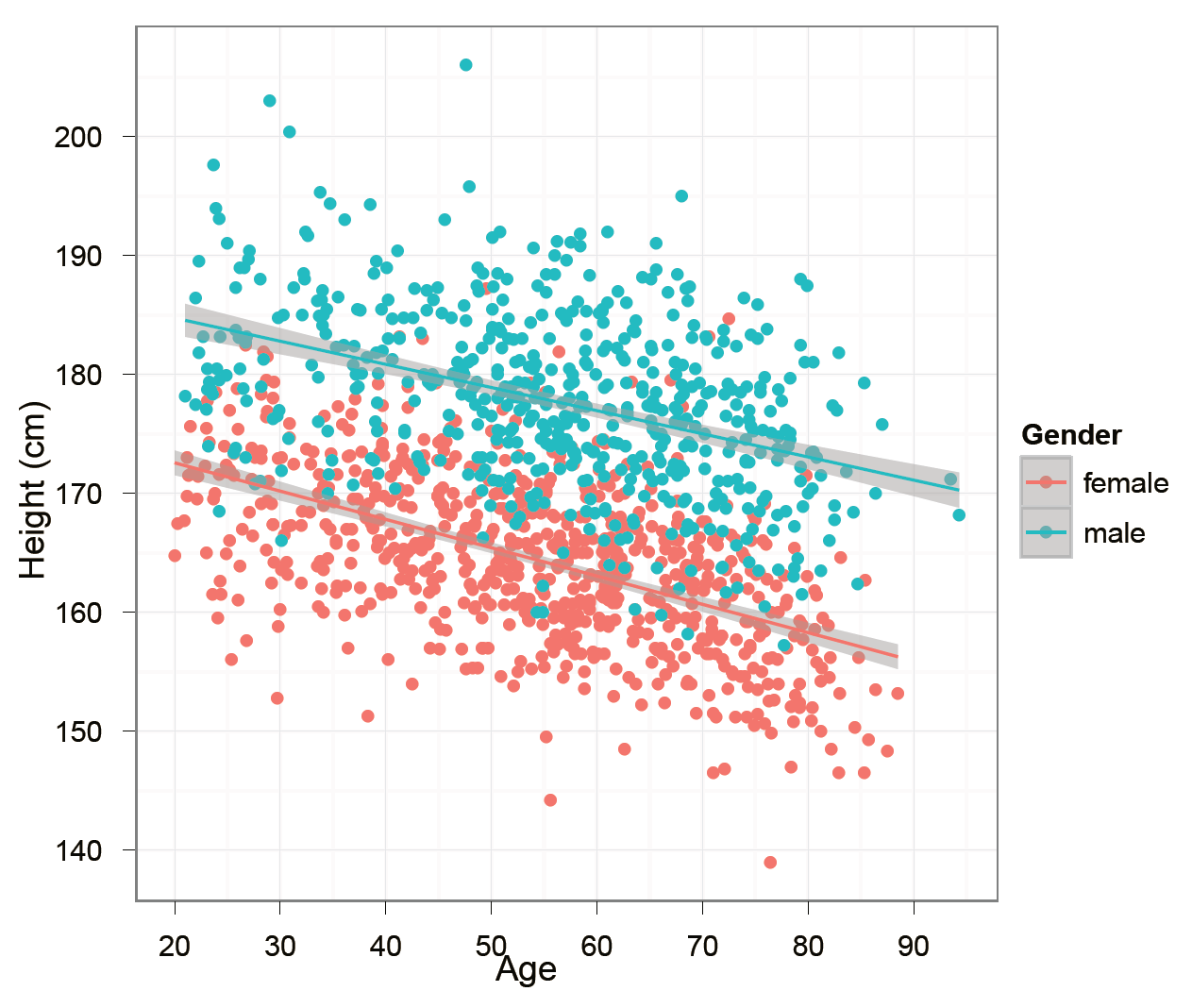

Supplement: Figure S1 — Correlation of height with age in males and females. (TIF) [file pone.0029475.s001.tif]

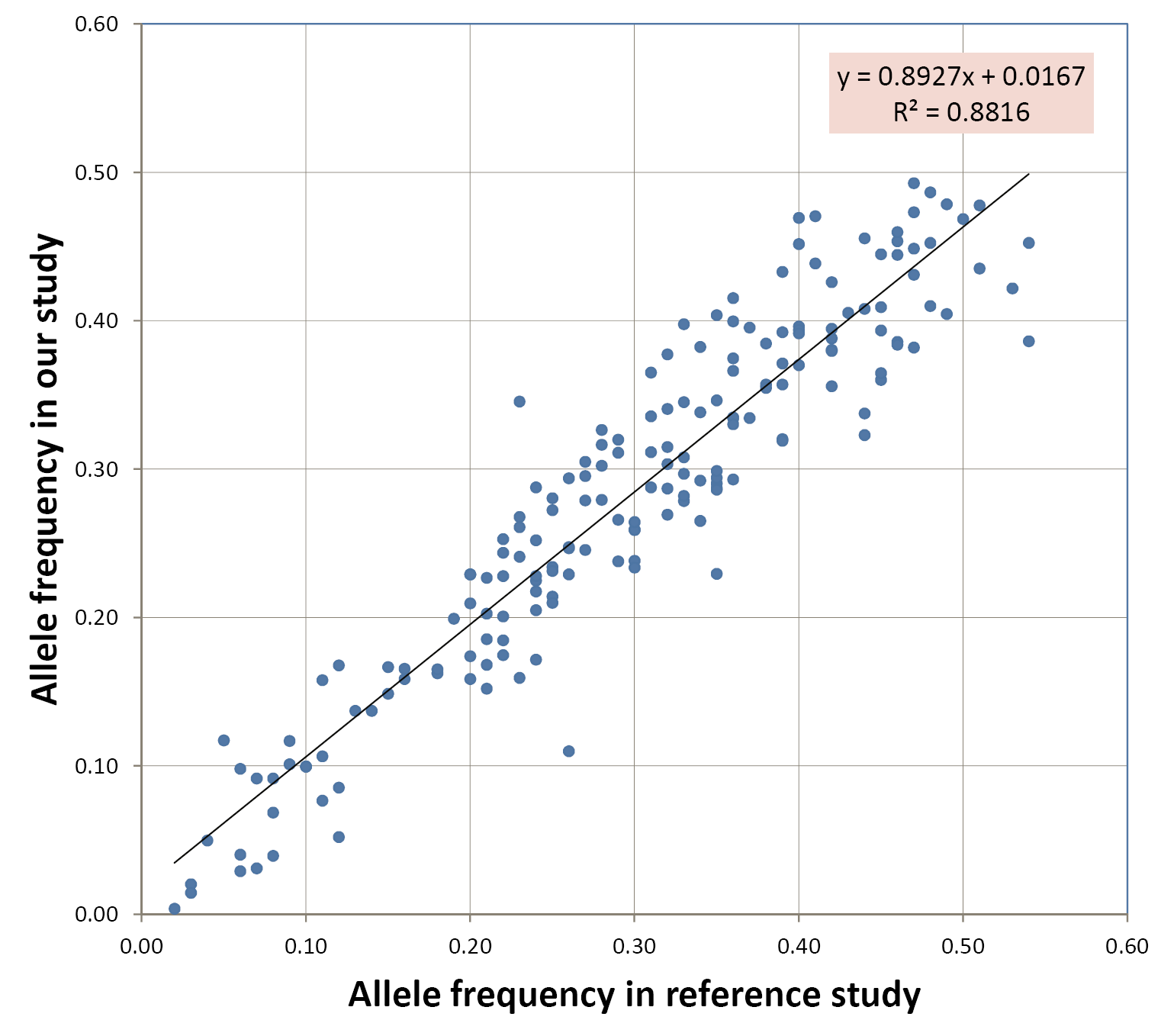

Supplement: Figure S2 — Minor allele frequencies of the 180 height-associated SNPs were highly correlated between our study and the reference study by Lango Allen et al [11] . (TIF) [file pone.0029475.s002.tif]

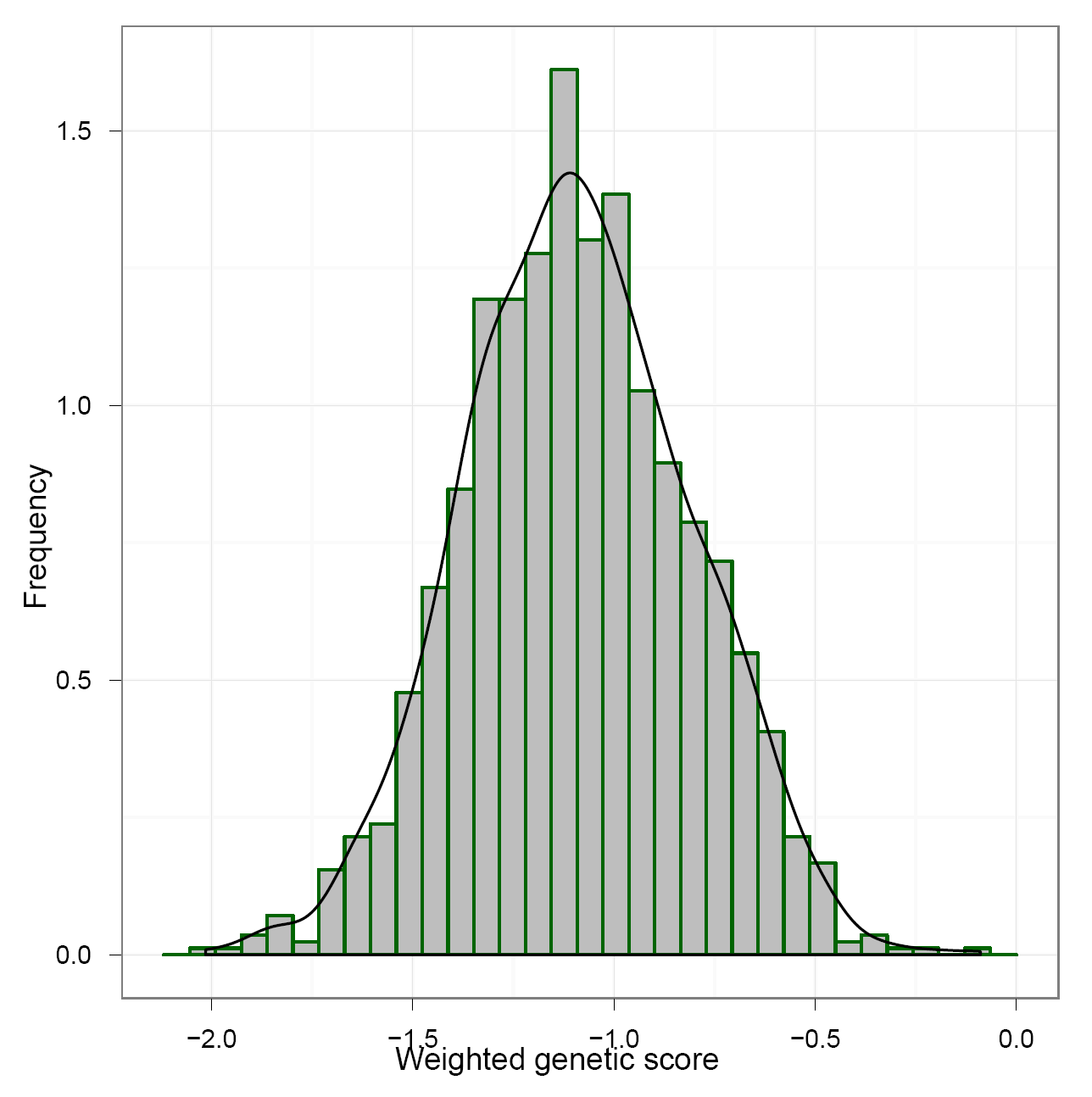

Supplement: Figure S3 — Genetic scores follow normal distribution. (TIF) [file pone.0029475.s003.tif]

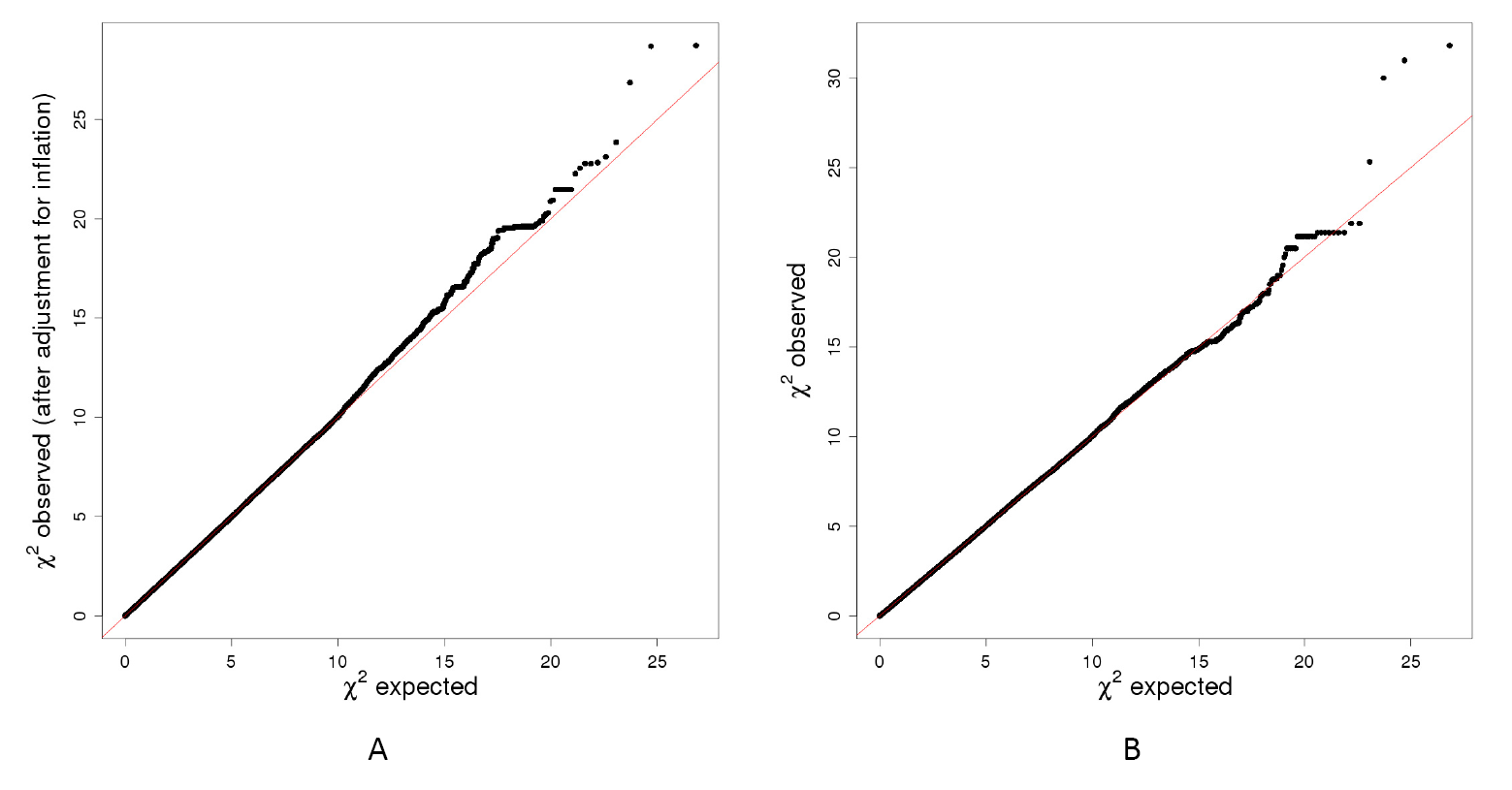

Supplement: Figure S4 — QQ plot of GC (A) and MG (B) association test. (TIF) [file pone.0029475.s004.tif]

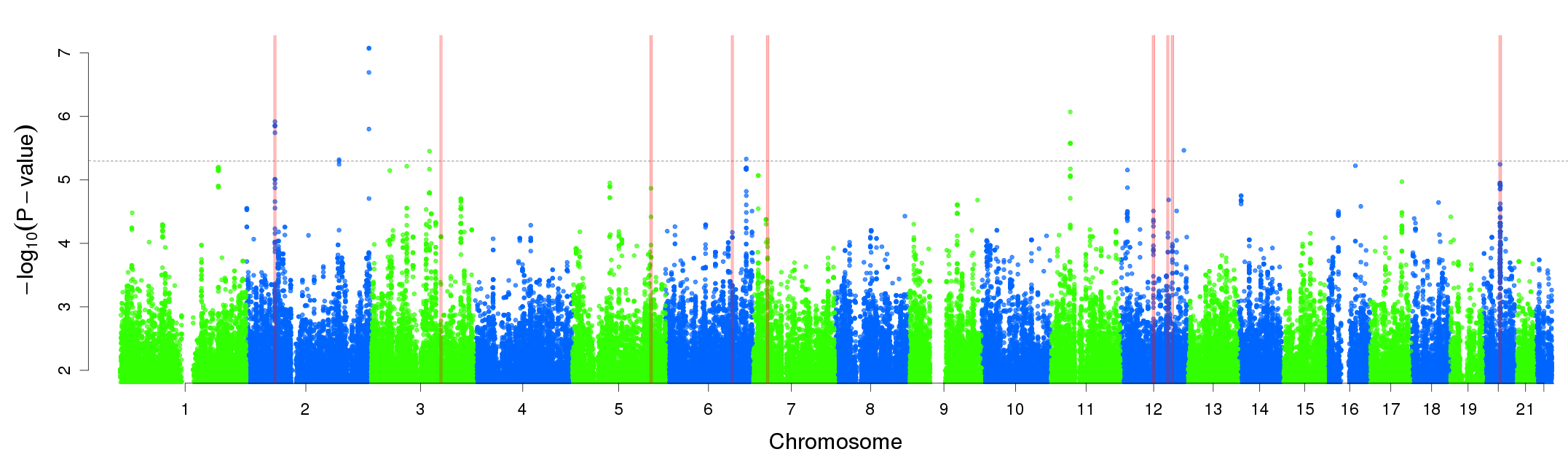

Supplement: Figure S5 — Manhattan plot (the red bars indicated the nine replicated GWA regions). (TIF) [file pone.0029475.s005.tif]
