# Supplementary material for: Extent of Height Variability Explained by Known Height-Associated Genetic Variants in an Isolated Population of the Adriatic Coast of Croatia
Source: PLoS One. 2011 Dec 27;6(12):e29475. doi: 10.1371/journal.pone.0029475 (PMC3246488; doi:10.1371/journal.pone.0029475)
Supplement: Table S1 — Frequencies and effect sizes of the 180 height-associated SNPs reported by Lango Allelen et al. [ Nature 467 (7317): 832-8]. (PDF) [file pone.0029475.s006.pdf]

Supplementary Table S1. Frequencies and effect sizes of the 180 height-associated SNPs reported by Lango Allelen et al. [*Nature* 467 (7317): 832-8]

SNP[@]	Chr	Position	Gene	A1	A2[#]	Reference study[$]			Our study[&]			P_{het}[*]
						Freq	Beta	P-value	Freq	Beta	P-value	
rs425277	1	2059032	*PRKCZ*	C	T	0.280	0.022	2.10E-08	0.302	-0.030	5.50E-01	0.305
rs2284746	1	17179262	*MFAP2*	G	C	0.480	-0.040	3.90E-29	0.487	-0.018	6.99E-01	0.628
rs1738475	1	23409478	*HTR1D*	C	G	0.410	-0.025	3.00E-12	0.470	0.035	4.58E-01	0.202
rs4601530	1	24916698	*CLIC4*	C	T	0.260	-0.028	2.20E-12	0.294	0.008	8.83E-01	0.495
rs7532866	1	26614131	*LIN28*	A	G	0.330	-0.021	3.40E-08	0.398	-0.012	8.05E-01	0.847
rs2154319	1	41518357	*SCMH1*	T	C	0.250	0.030	1.80E-12	0.280	0.044	3.94E-01	0.791
rs17391694	1	78396214	*GIPC2*	C	T	0.120	0.042	1.70E-11	0.052	0.106	3.07E-01	0.537
rs6699417	1	88896031	*PKN2*	T	C	0.390	-0.021	5.00E-09	0.357	-0.031	5.20E-01	0.841
rs10874746	1	93096559	*RPL5*	C	T	0.370	-0.024	6.70E-11	0.334	0.042	4.00E-01	0.185
rs9428104	1	1.19E+08	*SPAG17*	G	A	0.240	-0.041	5.60E-23	0.225	0.009	8.65E-01	0.352
rs11205277	1	1.48E+08	*SF3B4*	A	G	0.420	0.046	4.80E-32	0.356	0.121	2.59E-02	0.168
rs17346452	1	1.7E+08	*DNM3*	T	C	0.270	0.040	1.40E-23	0.246	0.063	2.31E-01	0.663
rs1325598	1	1.75E+08	*PAPPA2*	G	A	0.430	-0.022	1.10E-09	0.405	-0.041	3.83E-01	0.684
rs1046934	1	1.82E+08	*TSEN15*	A	C	0.360	0.044	2.10E-31	0.334	-0.072	1.37E-01	***0.017***
rs10863936	1	2.1E+08	*DTL*	A	G	0.470	0.021	1.90E-09	0.473	0.028	5.45E-01	0.881
rs6684205	1	2.17E+08	*TGFB2*	A	G	0.290	0.028	1.50E-12	0.266	0.133	1.01E-02	***0.043***
rs11118346	1	2.18E+08	*LYPLAL1*	C	T	0.470	-0.025	1.90E-12	0.449	-0.066	1.49E-01	0.373
rs10799445	1	2.26E+08	*JMJD4*	A	C	0.230	-0.032	2.40E-13	0.241	-0.069	2.06E-01	0.501
rs4665736	2	25041103	*DNAJC27*	T	C	0.460	-0.029	7.30E-16	0.454	-0.056	2.31E-01	0.562
rs6714546	2	33214929	*LTBP1*	G	A	0.280	-0.026	1.60E-09	0.279	-0.060	2.62E-01	0.527
rs17511102	2	37814117	*CDC42EP3*	A	T	0.090	0.060	1.60E-18	0.101	0.019	8.07E-01	0.597
rs2341459	2	44621706	*C2orf34*	C	T	0.270	0.025	7.90E-10	0.279	0.030	5.63E-01	0.926
rs12474201	2	46774789	*SOCS5*	G	A	0.350	0.028	2.60E-13	0.290	-0.027	5.84E-01	0.270
rs3791675	2	55964813	*EFEMP1*	C	T	0.230	-0.053	2.50E-35	0.346	-0.147	2.45E-03	0.054
rs11684404	2	88705737	*EIF2AK3*	T	C	0.330	0.028	9.90E-14	0.297	-0.015	7.67E-01	0.395
rs7567288	2	1.34E+08	*NCKAP5*	T	C	0.200	0.032	2.10E-12	0.159	-0.006	9.22E-01	0.542
rs7567851	2	1.78E+08	*PDE11A*	G	C	0.080	0.037	3.30E-08	0.092	0.088	2.79E-01	0.533
rs1351164	2	2.18E+08	*TNS1*	T	C	0.210	-0.034	2.10E-14	0.227	0.034	5.30E-01	0.210
rs12470505	2	2.2E+08	*CCDC108/IHH*	T	G	0.100	-0.041	8.90E-12	0.100	-0.107	1.64E-01	0.392
rs2629046	2	2.25E+08	*SERPINE2*	T	C	0.450	-0.024	7.90E-12	0.445	0.038	4.08E-01	0.178
rs2580816	2	2.33E+08	*NPPC*	C	T	0.190	-0.045	5.80E-22	0.199	-0.089	1.19E-01	0.440
rs12694997	2	2.42E+08	*SEPT2*	G	A	0.240	-0.024	1.20E-08	0.288	-0.087	8.42E-02	0.212
rs2597513	3	13530836	*HDAC11*	T	C	0.100	0.036	7.40E-10	0.100	0.039	6.19E-01	0.971
rs13088462	3	51046753	*DOCK3*	T	C	0.060	0.052	3.80E-10	0.098	0.158	3.78E-02	0.165
rs2336725	3	53093779	*RTF1*	T	C	0.450	0.027	9.70E-13	0.409	0.076	1.05E-01	0.297
rs9835332	3	56642722	*C3orf63*	G	C	0.460	-0.026	5.30E-13	0.386	-0.115	1.32E-02	0.056
rs17806888	3	67499012	*SUCLG2*	T	C	0.120	-0.036	2.10E-09	0.168	-0.002	9.75E-01	0.589
rs9863706	3	72520103	*RYBP*	C	T	0.220	-0.031	4.10E-13	0.185	-0.078	1.99E-01	0.440
rs6439167	3	1.31E+08	*C3orf47*	C	T	0.210	-0.034	8.90E-15	0.203	0.013	8.24E-01	0.415
rs9844666	3	1.37E+08	*PCCB*	G	A	0.250	-0.024	3.50E-09	0.214	0.045	4.17E-01	0.215
rs724016	3	1.43E+08	*ZBTB38*	A	G	0.440	0.070	3.10E-86	0.323	0.088	6.50E-02	0.711
rs572169	3	1.74E+08	*GHSR*	C	T	0.310	0.033	2.80E-18	0.311	0.039	4.26E-01	0.898
rs720390	3	1.87E+08	*IGF2BP2*	G	A	0.390	0.029	1.90E-14	0.320	-0.030	5.54E-01	0.242
rs2247341	4	1671115	*SLBP/FGFR3*	G	A	0.360	0.025	1.50E-11	0.293	0.069	1.71E-01	0.385
rs6449353	4	17642586	*LCORL*	T	C	0.150	-0.075	7.10E-46	0.167	-0.016	7.94E-01	0.346
rs17081935	4	57518233	*POLR2B*	C	T	0.200	0.030	3.70E-11	0.229	0.030	6.01E-01	0.995
rs7697556	4	73734177	*ADAMTS3*	C	T	0.470	0.028	2.00E-14	0.431	-0.014	7.63E-01	0.366
rs788867	4	82369030	*PRKG2/BMP3*	T	G	0.320	0.043	8.90E-28	0.377	0.102	3.02E-02	0.211

rs10010325	4	1.06E+08	*TET2*	A	C	0.510	-0.024	3.90E-11	0.478	0.055	2.36E-01	0.089
rs7689420	4	1.46E+08	*HHIP*	C	T	0.160	-0.073	6.20E-51	0.159	-0.065	2.90E-01	0.902
rs955748	4	1.84E+08	*WWC2*	G	A	0.240	-0.023	4.40E-08	0.205	0.052	3.61E-01	0.188
rs1173727	5	32866278	*NPR3*	C	T	0.400	0.034	1.60E-21	0.469	0.007	8.75E-01	0.555
rs11958779	5	55037656	*SLC38A9*	A	G	0.300	0.027	1.80E-12	0.259	0.050	3.48E-01	0.663
rs10037512	5	88390431	*MEF2C*	T	C	0.440	-0.032	2.00E-18	0.338	-0.050	3.06E-01	0.709
rs13177718	5	1.08E+08	*FER*	C	T	0.070	-0.040	3.00E-08	0.031	-0.082	5.36E-01	0.751
rs1582931	5	1.23E+08	*CEP120*	A	G	0.530	0.023	1.50E-10	0.422	0.039	4.11E-01	0.732
rs274546	5	1.32E+08	*SLC22A5*	G	A	0.400	-0.029	7.30E-16	0.452	0.032	4.98E-01	0.197
rs526896	5	1.34E+08	*PITX1*	T	G	0.270	-0.030	2.30E-13	0.295	-0.031	5.42E-01	0.985
rs4282339	5	1.68E+08	*SLIT3*	G	A	0.200	-0.036	6.60E-16	0.229	-0.016	7.67E-01	0.717
rs12153391	5	1.71E+08	*FBXW11*	C	A	0.250	-0.030	3.60E-12	0.234	-0.087	1.22E-01	0.313
rs889014	5	1.73E+08	*BOD1*	C	T	0.360	-0.030	9.40E-16	0.415	-0.070	1.38E-01	0.399
rs422421	5	1.76E+08	*FGFR4/NSD1*	C	T	0.220	-0.031	1.10E-12	0.244	0.004	9.40E-01	0.511
rs6879260	5	1.8E+08	*GFPT2*	C	T	0.390	-0.022	1.60E-09	0.392	-0.022	6.27E-01	0.992
rs3812163	6	7670759	*BMP6*	A	T	0.460	0.036	1.20E-23	0.460	0.038	4.07E-01	0.969
rs1047014	6	19949472	*ID4*	T	C	0.250	0.032	1.80E-13	0.231	0.032	5.52E-01	0.996
rs806794	6	26308656	*Histone cluster*	A	G	0.300	-0.052	1.20E-39	0.238	-0.125	1.99E-02	0.175
rs3129109	6	29192211	*OR2J3*	C	T	0.390	-0.032	2.40E-17	0.371	-0.022	6.47E-01	0.836
rs2256183	6	31488508	*MICA*	G	A	0.450	0.040	7.80E-29	0.360	0.086	7.40E-02	0.338
rs6457620	6	32771977	*HLA locus*	C	G	0.490	0.029	2.10E-16	0.405	0.026	5.78E-01	0.958
rs2780226	6	34307070	*HMGA1*	T	C	0.080	0.076	8.10E-28	0.039	-0.018	8.83E-01	0.434
rs6457821	6	35510783	*PPARD/FANCE*	C	A	0.020	-0.104	2.10E-12	0.004	0.303	4.18E-01	0.276
rs9472414	6	45054484	*SUPT3H/RUNX2*	T	A	0.220	-0.026	1.80E-09	0.253	-0.037	4.88E-01	0.839
rs9360921	6	76322362	*SENP6*	T	G	0.110	0.042	2.60E-13	0.107	-0.013	8.64E-01	0.472
rs310405	6	81857081	*FAM46A*	A	G	0.480	-0.026	2.20E-13	0.410	0.015	7.47E-01	0.378
rs7759938	6	1.05E+08	*LIN28B*	T	C	0.320	0.045	8.30E-31	0.341	-0.004	9.29E-01	0.298
rs1046943	6	1.1E+08	*ZBTB24*	A	G	0.420	-0.020	2.50E-08	0.395	-0.038	4.23E-01	0.702
rs961764	6	1.18E+08	*VGLL2*	G	C	0.420	-0.024	1.30E-11	0.426	-0.043	3.57E-01	0.688
rs1490384	6	1.27E+08	*C6orf173*	T	C	0.500	-0.034	3.90E-21	0.469	-0.059	1.91E-01	0.578
rs6569648	6	1.3E+08	*L3MBTL3*	T	C	0.240	0.040	1.10E-21	0.218	0.101	6.35E-02	0.263
rs7763064	6	1.43E+08	*GPR126*	G	A	0.290	-0.048	1.10E-33	0.320	-0.129	9.98E-03	0.106
rs543650	6	1.52E+08	*ESR1*	G	T	0.400	-0.034	1.20E-17	0.396	-0.055	3.38E-01	0.718
rs9456307	6	1.59E+08	*TULP4*	T	A	0.060	-0.048	2.20E-09	0.040	-0.018	8.76E-01	0.797
rs798489	7	2768329	*GNA12*	C	T	0.300	-0.048	1.90E-33	0.234	-0.087	1.04E-01	0.471
rs4470914	7	19583047	*TWISTNB*	C	T	0.180	0.029	9.20E-10	0.162	-0.040	5.20E-01	0.271
rs12534093	7	23469499	*IGF2BP3*	T	A	0.220	-0.034	2.00E-14	0.175	-0.067	2.67E-01	0.587
rs1708299	7	28156471	*JAZF1*	G	A	0.300	0.040	5.80E-25	0.259	-0.015	7.76E-01	0.288
rs6959212	7	38094851	*STARD3NL*	C	T	0.320	-0.024	1.60E-09	0.303	-0.010	8.50E-01	0.785
rs42235	7	92086012	*CDK6*	C	T	0.310	0.057	7.70E-47	0.365	0.069	1.47E-01	0.795
rs822552	7	1.48E+08	*PDIA4*	C	G	0.260	0.025	2.60E-08	0.110	0.020	7.89E-01	0.949
rs2110001	7	1.5E+08	*TMEM176A*	C	G	0.310	0.031	3.30E-13	0.336	0.106	3.13E-02	0.129
rs1013209	8	24172249	*ADAM28*	C	T	0.250	-0.025	1.60E-09	0.210	0.065	2.62E-01	0.122
rs7460090	8	57356717	*SDR16C5*	T	C	0.130	-0.058	8.20E-27	0.137	-0.014	8.33E-01	0.516
rs6473015	8	78341040	*PEX2*	A	C	0.280	0.029	6.90E-13	0.326	0.033	5.03E-01	0.935
rs6470764	8	1.31E+08	*GSDMC*	C	T	0.200	-0.050	1.70E-28	0.174	-0.029	6.27E-01	0.722
rs12680655	8	1.36E+08	*ZFAT*	C	G	0.400	-0.028	1.60E-14	0.394	-0.028	5.55E-01	1.000
rs7864648	9	16358732	*BNC2*	G	T	0.320	0.022	2.10E-08	0.287	0.019	7.07E-01	0.955
rs11144688	9	77732106	*PCSK5*	G	A	0.110	-0.049	9.60E-12	0.158	0.000	9.96E-01	0.430
rs7853377	9	85742025	*C9orf64*	A	G	0.230	0.024	4.50E-08	0.268	0.026	6.16E-01	0.968
rs8181166	9	88306448	*ZCCHC6*	C	G	0.470	-0.026	2.70E-12	0.493	0.017	7.04E-01	0.341
rs2778031	9	90025546	*SPIN1*	C	T	0.240	0.031	9.00E-13	0.252	0.011	8.30E-01	0.710
rs9969804	9	94468941	*IPPK*	C	A	0.440	0.030	7.70E-17	0.408	0.036	4.39E-01	0.903
rs1257763	9	95933766	*PTPDC1*	G	A	0.040	0.069	9.90E-10	0.050	0.299	3.92E-03	***0.027***

rs473902	9	97296056	*PTCH1/FANCC*	T	G	0.080	-0.065	2.30E-17	0.069	0.040	6.61E-01	0.253
rs7027110	9	1.09E+08	*ZNF462*	G	A	0.230	0.031	2.30E-13	0.159	-0.089	1.54E-01	0.055
rs1468758	9	1.13E+08	*LPAR1*	C	T	0.250	-0.026	1.40E-09	0.272	0.032	5.38E-01	0.265
rs751543	9	1.18E+08	*PAPPA*	T	C	0.280	-0.026	6.50E-10	0.316	-0.060	2.16E-01	0.484
rs7466269	9	1.32E+08	*FUBP3*	A	G	0.360	-0.032	2.60E-17	0.335	-0.008	8.75E-01	0.622
rs7849585	9	1.38E+08	*QSOX2*	G	T	0.330	0.029	4.70E-14	0.279	-0.035	4.77E-01	0.196
rs7909670	10	12958770	*CCDC3*	C	T	0.440	-0.021	3.20E-09	0.456	-0.031	5.05E-01	0.826
rs2145998	10	80791702	*PPIF*	A	T	0.510	0.026	3.60E-13	0.435	0.013	7.74E-01	0.781
rs11599750	10	1.02E+08	*CPN1*	C	T	0.380	-0.028	1.60E-13	0.357	0.010	8.36E-01	0.421
rs2237886	11	2767307	*KCNQ1*	C	T	0.110	0.046	2.20E-13	0.077	-0.014	8.69E-01	0.489
rs7926971	11	12654616	*TEAD1*	A	G	0.450	0.023	4.40E-10	0.393	-0.078	1.06E-01	***0.037***
rs1330	11	17272605	*NUCB2*	C	T	0.350	0.022	4.90E-09	0.230	-0.056	3.04E-01	0.153
rs10838801	11	48054856	*PTPRJ/SLC39A13*	A	G	0.310	0.027	3.50E-12	0.288	0.008	8.77E-01	0.713
rs1814175	11	49515748	*FOLH1*	C	T	0.340	0.022	1.60E-08	0.265	-0.067	2.05E-01	0.094
rs5017948	11	51270794	*OR4A5*	T	A	0.180	0.027	3.10E-08	0.165	-0.020	7.48E-01	0.452
rs3782089	11	65093395	*SSSCA1*	C	T	0.060	-0.058	3.60E-13	0.029	-0.033	8.13E-01	0.857
rs7112925	11	66582736	*RHOD*	C	T	0.350	-0.023	9.00E-10	0.404	0.000	9.96E-01	0.638
rs634552	11	74959700	*SERPINH1*	G	T	0.140	0.039	3.50E-13	0.137	0.057	4.13E-01	0.798
rs494459	11	1.18E+08	*TREH*	C	T	0.410	0.020	1.70E-08	0.439	-0.042	3.58E-01	0.176
rs654723	11	1.28E+08	*FLI1*	A	C	0.380	-0.025	3.60E-11	0.355	0.080	9.21E-02	***0.027***
rs2856321	12	11747040	*ETV6*	A	G	0.360	0.029	4.50E-15	0.366	0.012	8.05E-01	0.725
rs10770705	12	20748734	*SLCO1C1*	C	A	0.330	0.033	8.00E-18	0.282	0.087	9.54E-02	0.302
rs2638953	12	28425682	*CCDC91*	C	G	0.320	-0.032	6.70E-17	0.269	-0.043	4.19E-01	0.836
rs2066807	12	55026949	*STAT2*	C	G	0.070	0.054	1.00E-13	0.092	-0.067	3.97E-01	0.127
rs1351394	12	64638093	*HMGA2*	C	T	0.490	0.060	1.70E-65	0.479	0.171	1.60E-04	***0.015***
rs10748128	12	68113925	*FRS2*	G	T	0.350	0.038	2.10E-20	0.287	-0.057	2.57E-01	0.060
rs11107116	12	92502635	*SOCS2*	G	T	0.220	0.052	1.40E-34	0.228	0.095	7.97E-02	0.428
rs7971536	12	1.01E+08	*CCDC53/GNPTAB*	T	A	0.460	-0.028	8.20E-14	0.444	-0.015	7.48E-01	0.774
rs11830103	12	1.22E+08	*SBNO1*	A	G	0.220	0.035	3.90E-15	0.201	-0.033	5.66E-01	0.238
rs7332115	13	32045548	*PDS5B/BRCA2*	T	G	0.380	0.023	5.50E-10	0.385	0.064	1.68E-01	0.378
rs3118905	13	50003335	*DLEU7*	G	A	0.290	-0.056	1.10E-45	0.311	0.093	6.32E-02	***0.003***
rs7319045	13	90822575	*GPC5*	G	A	0.400	0.025	1.20E-11	0.370	-0.015	7.47E-01	0.395
rs1950500	14	23900690	*NFATC4*	C	T	0.290	0.034	2.20E-18	0.238	0.011	8.39E-01	0.673
rs2093210	14	60027032	*SIX6*	T	C	0.420	0.032	6.20E-17	0.388	0.030	5.30E-01	0.958
rs1570106	14	67882868	*RAD51L1*	C	T	0.200	-0.026	8.10E-09	0.210	0.008	8.89E-01	0.554
rs862034	14	74060499	*LTBP2*	G	A	0.360	-0.028	7.30E-14	0.400	-0.040	3.87E-01	0.794
rs7155279	14	91555634	*TRIP11*	G	T	0.360	-0.024	1.40E-10	0.330	-0.035	4.57E-01	0.814
rs16964211	15	49317787	*CYP19A1*	G	A	0.050	-0.050	1.70E-09	0.117	-0.016	8.29E-01	0.637
rs7178424	15	60167551	*C2CD4A*	C	T	0.470	-0.021	5.60E-09	0.382	0.029	5.40E-01	0.293
rs10152591	15	67835211	*TLE3*	A	C	0.090	-0.041	2.70E-10	0.117	0.026	7.19E-01	0.356
rs12902421	15	69948457	*MYO9A*	T	C	0.030	0.062	2.90E-08	0.020	0.164	3.21E-01	0.538
rs5742915	15	72123686	*PML*	C	T	0.540	-0.031	1.00E-15	0.386	-0.187	3.42E-04	***0.003***
rs11259936	15	82371586	*ADAMTSL3*	C	A	0.480	-0.044	1.70E-35	0.452	-0.081	8.50E-02	0.430
rs16942341	15	87189909	*ACAN*	C	T	0.030	-0.130	3.80E-27	0.015	-0.355	6.65E-02	0.245
rs2871865	15	97012419	*IGF1R*	C	G	0.120	-0.057	2.90E-21	0.085	-0.145	6.85E-02	0.271
rs4965598	15	98577137	*ADAMTS17*	T	C	0.320	0.028	4.30E-13	0.315	0.045	3.42E-01	0.720
rs11648796	16	732191	*NARFL*	A	G	0.260	0.034	1.20E-13	0.247	0.039	4.75E-01	0.934
rs26868	16	2189377	*CASKIN1*	A	T	0.540	-0.034	9.00E-17	0.452	-0.112	2.95E-02	0.131
rs1659127	16	14295806	*MKL2*	G	A	0.340	0.027	1.10E-11	0.382	0.029	5.40E-01	0.964
rs8052560	16	87304743	*CTU2/GALNS*	A	C	0.210	-0.029	3.30E-08	0.185	-0.053	3.82E-01	0.691
rs4640244	17	21224816	*KCNJ12*	A	G	0.390	-0.024	2.30E-08	0.319	0.115	3.67E-02	***0.012***
rs3110496	17	24941897	*ANKRD13B*	G	A	0.330	-0.022	7.30E-09	0.345	-0.042	3.98E-01	0.690
rs3764419	17	26188149	*ATAD5/RNF135*	C	A	0.390	-0.035	1.80E-21	0.433	-0.088	6.12E-02	0.262
rs17780086	17	27367395	*LRRC37B*	G	A	0.150	0.028	2.60E-08	0.149	0.007	9.15E-01	0.748

rs1043515	17	34175722	*PIP4K2B*	G	A	0.450	-0.023	2.90E-10	0.365	0.009	8.50E-01	0.502
rs4986172	17	40571807	*ACBD4*	C	T	0.350	-0.032	2.30E-16	0.294	0.090	7.47E-02	***0.016***
rs2072153	17	44745013	*ZNF652*	G	C	0.300	0.021	3.50E-08	0.264	-0.007	8.99E-01	0.601
rs4605213	17	46599746	*NME2*	G	C	0.340	0.021	2.70E-08	0.338	0.010	8.44E-01	0.815
rs227724	17	52133816	*NOG*	A	T	0.350	0.030	7.40E-15	0.286	-0.024	6.27E-01	0.276
rs2079795	17	56851431	*TBX2*	C	T	0.330	0.040	2.10E-24	0.308	0.035	4.67E-01	0.925
rs2665838	17	59320197	*CSH1/GH1*	C	G	0.270	0.042	5.10E-25	0.305	0.051	3.18E-01	0.861
rs11867479	17	65601802	*KCNJ16/KCNJ2*	C	T	0.340	0.025	1.50E-10	0.292	-0.017	7.43E-01	0.418
rs4800452	18	18981609	*CABLES1*	T	C	0.210	-0.051	4.20E-30	0.152	-0.138	2.94E-02	0.171
rs9967417	18	45213498	*DYM*	C	G	0.420	0.038	9.30E-25	0.380	-0.010	8.31E-01	0.315
rs17782313	18	56002077	*MC4R*	T	C	0.240	0.028	3.80E-11	0.228	0.069	1.96E-01	0.443
rs12982744	19	2128193	*DOT1L*	C	G	0.400	0.030	3.40E-16	0.392	0.009	8.55E-01	0.662
rs7507204	19	3379834	*NFIC*	G	C	0.240	0.036	4.30E-16	0.172	0.045	4.59E-01	0.883
rs891088	19	7135762	*INSR*	A	G	0.260	0.029	2.40E-12	0.248	0.098	7.25E-02	0.208
rs4072910	19	8550031	*ADAMTS10*	G	C	0.460	-0.031	3.60E-13	0.384	-0.041	5.55E-01	0.885
rs2279008	19	17144303	*MYO9B*	T	C	0.260	-0.025	2.50E-08	0.229	-0.146	1.57E-02	***0.046***
rs17318596	19	46628935	*ATP5SL*	G	A	0.360	0.032	5.00E-16	0.375	0.113	2.47E-02	0.109
rs1741344	20	4049800	*SMOX*	T	C	0.370	0.023	3.30E-09	0.395	0.082	7.56E-02	0.203
rs2145272	20	6574218	*BMP2*	A	G	0.350	0.039	2.10E-24	0.346	0.074	1.26E-01	0.471
rs7274811	20	31796842	*ZNF341*	G	T	0.230	-0.041	5.90E-22	0.261	0.027	6.09E-01	0.196
rs143384	20	33489170	*GDF5*	A	G	0.420	0.063	1.00E-58	0.380	0.161	8.09E-04	***0.042***
rs237743	20	47336426	*ZNFX1*	G	A	0.210	0.041	1.30E-20	0.168	0.141	2.15E-02	0.104
rs2834442	21	34612656	*KCNE2*	A	T	0.350	-0.026	5.10E-12	0.299	-0.037	4.67E-01	0.832
rs4821083	22	31386341	*SYN3*	T	C	0.160	-0.031	3.10E-10	0.166	0.013	8.36E-01	0.479

[@] The 180 height-associated SNPs and their chromosome positions and gene annotations were obtained from Supplementary Table 1 of Lango Allelen et al. [*Nature* 467 (7317): 832-8]

[#] The major (**A1**) and the minor (**A2**) alleles were determined based on the observed allele frequencies in our study samples. The minor alleles (**A2**) were selected as reference alleles.

[$] The effect sizes and p-values of the reference study were extracted from the column [STAGE 1+ STAGE 2] of Supplementary Table 1 of Lango Allelen et al. The direction of effects and the allele frequencies were reversed if our reference alleles (**A2**) are different from the original table.

[&] The effect sizes were estimated from our study samples (*N*=1304) and the p-values were genomic control (GC) adjusted 1df p-values by an inflation factor (λ=1.41).

[*] The heterogeneity of effect sizes between the reference study and our study was tested using z-test. P-values reaching nominal significance ($P_{het} < 0.05$) were highlighted in italic.
